# Design and Experimental Validation of a 3D-Printed Embedded-Sensing Continuum Robot for Neurosurgery

**DOI:** 10.3390/mi14091743

**Published:** 2023-09-06

**Authors:** Donatella Dragone, Francesca Federica Donadio, Chiara Mirabelli, Carlo Cosentino, Francesco Amato, Paolo Zaffino, Maria Francesca Spadea, Domenico La Torre, Alessio Merola

**Affiliations:** 1 Dipartimento di Ingegneria Elettrica e delle Tecnologie dell’Informazione, Università degli Studi di Napoli Federico II, Via Claudio 21, 80125 Napoli, Italy; donatella.dragone@unicz.it (D.D.);; 2 Biomechatronics Laboratory, Department of Experimental and Clinical Medicine, Università degli Studi Magna Græcia di Catanzaro, Campus Universitario “S. Venuta”, 88100 Catanzaro, Italy; 3 Department of Medical and Surgical Sciences, Università degli Studi Magna Græcia di Catanzaro, Campus Universitario “S. Venuta”, 88100 Catanzaro, Italy; dlatorre@unicz.it

**Keywords:** hyper-redundant manipulator, optoelectronic sensor, medical robotics, multiple regression model

## Abstract

A minimally-invasive manipulator characterized by hyper-redundant kinematics and embedded sensing modules is presented in this work. The bending angles (tilt and pan) of the robot tip are controlled through tendon-driven actuation; the transmission of the actuation forces to the tip is based on a Bowden-cable solution integrating some channels for optical fibers. The viability of the real-time measurement of the feedback control variables, through optoelectronic acquisition, is evaluated for automated bending of the flexible endoscope and trajectory tracking of the tip angles. Indeed, unlike conventional catheters and cannulae adopted in neurosurgery, the proposed robot can extend the actuation and control of snake-like kinematic chains with embedded sensing solutions, enabling real-time measurement, robust and accurate control of curvature, and tip bending of continuum robots for the manipulation of cannulae and microsurgical instruments in neurosurgical procedures. A prototype of the manipulator with a length of 43 mm and a diameter of 5.5 mm has been realized via 3D printing. Moreover, a multiple regression model has been estimated through a novel experimental setup to predict the tip angles from measured outputs of the optoelectronic modules. The sensing and control performance has also been evaluated during tasks involving tip rotations.

## 1. Introduction

An important technological breakthrough in neurosurgery can be potentially achieved through the integration of minimally-invasive robots within intraoperative magnetic resonance imaging (MRI) systems. Indeed, such systems can help the surgeon to localize the MRI-compatible surgical robot for more accurate intraoperative control of the robot’s trajectory in a constrained space. Robots having multiple degrees-of-freedom (DoF) with MRI-compatible features potentially enable a complete removal of a tumor with accurate tracking performance, within the framework of novel deep learning algorithms for the elaboration of intraoperative magnetic resonance images [1] allowing the accurate and precise localization of polymeric catheters.

The conventional design of neurosurgical instruments may limit the efficacy, e.g., of the evacuation of high hematoma, especially when important brain structures are located along the desired straight-line access path and peripheral areas of semi-coagulated blood clots that need to be reached [2]. To overcome the main limitations of the standard instrumentation, the overall prototype development, which is presented here in its first stages, is focused on the design, realization, and testing of a novel neurosurgical robot belonging to the class of continuum robots.

The starting point of the design process has been the choice and optimization of the kinematic chain and sensing modules of the robot, in order to confer enhanced workspace and dexterity to the implementation of the closed-loop control of the tip orientation within a continuum kinematics. Conveniently, the robot prototype can take a tubular design hosting conduits for intra-cerebral suction and irrigation, as well as channels for sensing, tendon-based actuation, and microsurgical instruments for tumor removal.

As in general procedures of single-port surgery [3,4], a continuum robot can be inserted via intracranial access and directed towards the targeted surgical site. A number of neurosurgical tasks can be performed by deploying miniaturized instruments at the robot tip. In addition, robot instrumentation should benefit from inexpensive and disposable solutions for sterile operation. To this end, both surgical tools and actuation mechanisms should be of moderate complexity, with tendons transferring the actuation inputs from the proximal to the distal portions of the robot [5].

The extensive research on continuum robots, which has been carried out in the past few decades [6,7], has highlighted many advantages arising from the flexible structure of such manipulators. Some relevant applications have been tested (see, e.g., [8,9]) towards the development of robot-aided neurosurgery, providing the possibility of controlling the shape of the robot body and its distal portion during its interaction with biological tissues, including the control of actuated needles. More recently, a continuum robotic cannula consisting of a pre-curved tube and a cable-driven dexterous tip has been proposed in [2]. The unique and advanced design of this continuum robot provides distal dexterity with a continuous lumen, which can be exploited to achieve simultaneously curved motion for instrument insertion and distal dexterity for optimal hemorrhage evacuation.

Continuum robots can meet the design requirements for distal dexterity in a confined workspace, safe tissue–robot interaction, and disposability thanks to their compliant body which can be cable-driven by actuators placed at the proximal end of the robot (see, e.g., [10,11]). More importantly, the flexible hardware of the robot can be easily obtained through the adoption of a series of rolling joints and elastic structural modules. The ease of fabrication through additive manufacturing techniques can be assured by elastic structures which can be 3D-modelled and printed as tubes with notch patterning. Alternatively, flexible structures characterized by lateral notches can be realized by cutting superelastic Nitinol tubes. Such a design has been adopted for improving the bendability and distal dexterity of neuroendoscopic (see [12,13]) and skull surgical instruments [14].

The flexible structure, which is obtained through the interconnection of several robotic modules, can enhance distal dexterity. However, the control of the curvature and bending of the distal portion of the robot becomes more complex. Therefore, accurate control cannot do without an even more accurate measurement of feedback variables.

Even though open-loop control laws have been proposed for the control of continuum manipulators, i.e., exploiting kinematic models for regulating the cable actuation lengths to achieve a desired curvature of the robot body or cable tension on the basis of dynamics models (see [15,16,17]), there is a lack of approaches for closed-loop control. This is because the embedding of bending/shape sensing modules into the structure of a continuum robot is not straightforward, especially in the presence of tight constraints on miniaturization required by general applications of robot-aided minimally-invasive surgery. Only recently have electromagnetic sensing modules been integrated into a hand-held robotic endoscope [18]. That work implements, in a closed-loop scheme, two-mode joystick control and a robot-arm admittance control with gravity compensation for coordinating a standalone hand-held endoscope and a robot-arm assisted endoscope.

The control of the curvature radius of a continuum manipulator has been evaluated in [19], achieving a mean error of 2 mm. Some permanent magnets are embedded into the robot body in order to detect the change of shape of the manipulator.

Electromagnetic sensors are not a viable solution whenever tight constraints on disposability, MRI-compatibility, and miniaturization are contemplated in the design process. The embedding of sensors within the robot structure can provide the proprioceptive channels required to estimate the robot shape with adequate accuracy for closed-loop control while keeping a compact and tubular design that is appealing for minimally-invasive surgical applications. A review of recent technologies and applications of shape sensing in medical robotics is provided in [20].

Inertial and electromagnetic sensors have been validated for shape sensing and accurate motion control of continuum manipulators. The feasibility of the implementation through optical-based measurements of shape sensing has been recently evaluated in [21] for snake-like manipulators. Optical fiber cables are embedded along the sections of the manipulator axis and the light reflected at each section is conveyed to the phototransistors installed on the base of the robot. More sophisticated techniques for shape sensing are those based on fiber Bragg gratings (FBGs). Inside optical fibers, some gratings are engraved and fused into the fiber diameter. The bending of the optical grating can be transduced into a modulated wavelength of the light signal through the fiber. Shape sensing solutions in the tridimensional space of tendon-driven continuum robots (see [22,23]) have provided an accurate estimation of the robot shape, together with other advantages in terms of miniaturization, biocompatibility, and MRI compatibility. On the other hand, some imperfections in the manufacturing process of FBGs can negatively impact shape estimation accuracy. Moreover, an accurate calibration is required to compensate for temperature drift and fiber torsion whenever the support structure of FBG is not sufficiently rigid. Additionally, the sophisticated optical setup required to transduce shape changes is not cost-effective.

Flexible and stretchable optical waveguides [24,25,26] have been embedded into soft pneumatic actuators to measure the bending of the actuator body. In the literature on Soft Robotics, optical waveguides are referred to as interesting solutions for the optical sensorization of actuators obtained through the fabrication of soft optical channels integrated into compliant actuators.

A much more interesting and advanced setup for the bending measurement and contact force sensing of soft robots is presented in [27], where the utilization of rigid optical fibers, instead of soft ones, enables the installation of all the optoelectronic components on the distal portion of the actuator, whereas the actuation module is placed on the proximal side of the device. Advantageously, some rigid optical fibers guarantee the transmission of optical signals over long distances; the resulting setup separates the optoelectronic components, i.e., light-emitting diode and photodiode, at a distance from the actuator itself. The embedded optical fibers, which are inflected during the bending motion, make the proprioceptive channel exploitable for bending measurements. The optical signal travels the pathway inside the actuator provided by the fiber channel connecting the light source and the receiver; the optical pathway, along the axis of the actuator, is U-shaped since it starts from the proximal portion of the actuator and then, after reaching the actuator tip, it comes back to the proximal section hosting the optoelectronic components. Through the attenuation of the optical signal acquired on the receiver side, it is possible to obtain the bending measurement. A similar optical setup, together with non-conductive and non-magnetic properties of the main components of soft pneumatic actuators, can meet the requirements for electromagnetic interference and MRI compatibility.

The adoption of rolling joints within the kinematic chain of a cable-driven continuum robot can provide unique advantages in terms of improved motion/interaction control, thanks to the modality of regulation of the cable tension, enhanced dexterity, and optimized bending and orientation of the robot tip. In the present work, the extension of the advantages of the rolling-joints kinematics to the efficient control of minimally-invasive and MRI-compatible neurosurgical robots has motivated the development and testing of a novel cable-driven continuum manipulator, which is also 3D-printable. To the best of the authors’ knowledge, this work presents for the first time a solution of embedded sensing for the miniaturized integration of optical-fiber channels within a continuum kinematics based on rolling joints. Therefore, the first goal of the prototype development is to evaluate the feasibility of the real-time measurement of the bending and orientation at the distal extremity of the robot body for purposes of closed-loop control.

The main phases of development and experimental testing of a first prototype of the manipulator, which are presented here, have involved a careful design of the setup of measurement and interface of the optical fibers providing the feedback signal of the orientation angles at the distal extremity of the robot, which is required for closed-loop control. The hyper-redundant and flexible manipulator is realized by interconnecting, in serial chain, seven sections through rolling joints and actuation cables. The resulting kinematic chain provides two DoFs for the tilt and pan motion of the distal extremity of the continuum manipulator (see [10] and Figure 6a therein). Moreover, the embedding within the manipulator body of optical sensing channels has required: (i) the optimization of the geometry and dimensions of the sections of the continuum manipulator, (ii) the integration with the mechanisms transducing the actuation forces into the cable tensions for bending control, (iii) the optimization of the optical setup and fiber wiring to reduce optical losses in correspondence with geometrical discontinuities within the robot sections.

The characterization of the measurement performance, which results from the embedded optoelectronic modules, has been evaluated on the basis of a multiple regression model. A series of experimental tests, which have been performed in different configurations within the workspace of the continuum manipulator, have been exploited to estimate the regression model which correlates the optoelectronic outputs with the two distal angles of the manipulator body. Moreover, both the sensing and control performances of the robot during tasks of orientation of its distal end have been evaluated.

This paper is structured as follows. First, Section 2 provides the design requirements, an overview of the actuation and control system of the continuum manipulator together with the discussion of the methodology and of the measurement principle of the distal bending angles. The experimental setup and the characterization of the optoelectronic measurement system are presented in Section 3. At last, the results analysis and conclusions are given in Section 4.

## 2. Materials and Methods

### 2.1. System Requirements and Overview

The continuum manipulator has a kinematic chain exploiting the advantages of the rolling joints, e.g., in terms of distal dexterity and payload capability arising from the optimized and tension-induced cable elongation.

To improve both the control accuracy and robustness against positioning errors related to cable elongation, the optical bending sensors are installed on-board along the continuum body of the robot to address some requirements on the closed-loop control of the bending and orientation of the distal extremity of the manipulator.

Robot-assisted neurosurgical tasks impose tight constraints, mainly in terms of safe interaction and dexterity in confined spaces. All the addressable design challenges can be summarized as follows.

**Figure 1:**
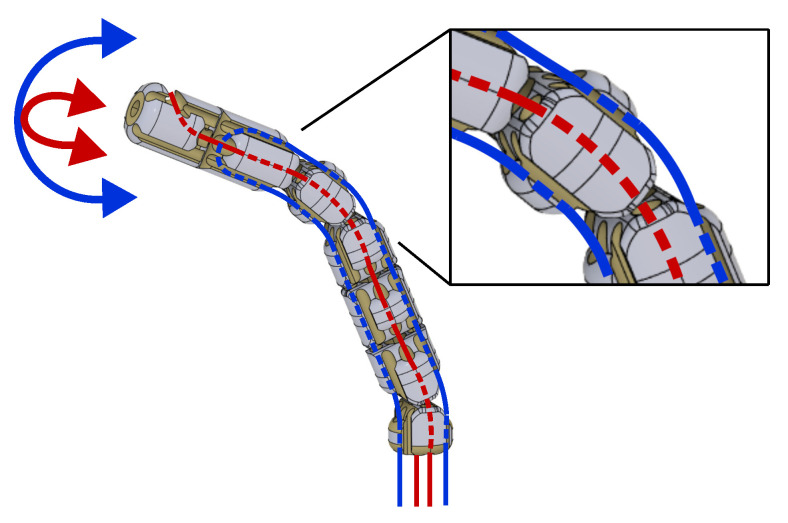
Three-dimensional (3D) model of the manipulator giving the details of both the rolling joints kinematics (zoomed view) and its DoF (distal rotations of tilt and pan, in blue and red, respectively).

(a) **Distal dexterity and tracking accuracy.** The cable-actuated continuum manipulator should guarantee a suitable range of ±45° around two independent axes on the distal extremity of the robot, which matches with the bending range of the realized prototype (see Figure 1). Through the optoelectronic sensing module of the robot body, the maximum measurement error in distal angles can be estimated through the experimental setup and procedure described in Section 3.
(b) **Safe interaction.** Variable-stiffness and improved cable tension control, which can be achieved through the adoption of a rolling-joints kinematics, can confer to the manipulator structure the controllability of the compliance required to limit the risk of damage to the biological tissues during positioning and manipulation of the continuum robot.
(c) **Miniaturization and workspace size.** A bending tip of length 43 mm has been judged adequate for accomplishing general neurosurgical tasks, including those performed in the presence of kinematic constraints within a small target area during hemorrhage aspiration and tumor resection. The diameter of the robot body should be comparable to those of conventional neurosurgical catheters of 5 mm.
(d) **Payload and accurate cabling design.** The force range required for surgical manipulation tasks, which has been estimated for endoscopic surgery, is [0.5–3] N [28]. The CAD-3D modeling involves a refined design of the conduits which, within the robot body, host the Bowden cable actuation and two U-shaped optical fibers. The path and curvature radius of such channels must be carefully designed to avoid slackness and breaking over the expected range of tension of the actuation cables.
(e) **Disposability and biocompatibility**. The cable-based solution enables the separation of the continuum robot from the actuation units. The robot can be 3D-printed through cost-effective (and bio-compatible) materials and, therefore, it can be easily disassembled and disposed.
(f) **MRI-compatibility**. All the sensing components and fabrication materials of the robot are MRI-compatible; moreover, the actuation unit can be installed far from the MRI volume for complying with MRI requirements.

### 2.2. Optical Sensing Unit

By embedding the optical fibers within the robot body, the propagation of the light through the fibers can vary depending on the body curvature, since the light flux has a lateral refraction over the curved paths and more light is lost under increasing bending. Therefore, the angles at the distal extremity of the robot, which are correlated to the overall curvature of the robot, can be estimated through a measure of the attenuation of the light transmitted through the fibers.

**Figure 2:**
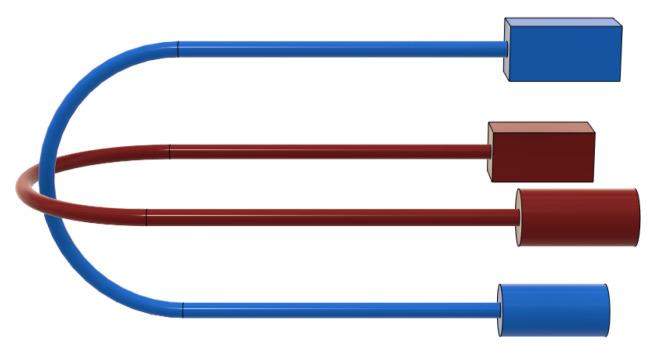
Scheme of the optical sensing unit. Two fibers on orthogonal planes within the robot body are used for the two DoFs corresponding to the distal rotations of tilt and pan.

**Figure 3:**
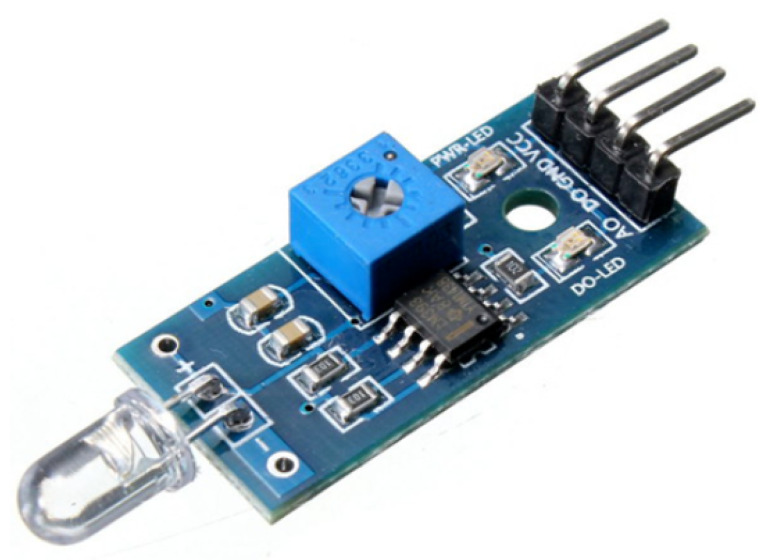
Receiver PCB of the optical sensing unit including a photodiode with LM393 amplifier.

The U-shaped optical fibers can bend, under antagonistic actuation, on two orthogonal planes contained within the body of the proposed continuum manipulator (see Figure 2). A commercial high-brightness LED is connected to one end of each fiber and it is used as a light source (represented by a cylinder in Figure 2); through an optical joint at the other extremity of the fiber, the receiver (represented by a box in Figure 2) allows the acquisition and transduction in an electric signal of the light transmitted through the fiber undergoing bending. The receiver board is based on commercially-available opto-electronic components (see Figure 3).

Each photodiode gives as output a voltage proportional to the attenuation of light due to bending. A microcontroller Arduino UNO R3 is used as an acquisition board. Other than the digital acquisition of the analog voltage of each photodiode in the range [0–5] V, the elaboration of the photodiode outputs is implemented on the Arduino board, since the output of each photodiode needs the compensation of a bias due to the non-null value of the photodiode voltage that is also in correspondence with a no-bending configuration of the continuum robot, i.e., in which the manipulator body is straight and the values of the distal angles are null. The voltage variations, which are obtained by compensating the readings of the photodiode voltage in the no-bending configuration, are used for identifying the multiple regression model used for estimating the distal angles from optoelectronic measurements.

**Figure 4:**
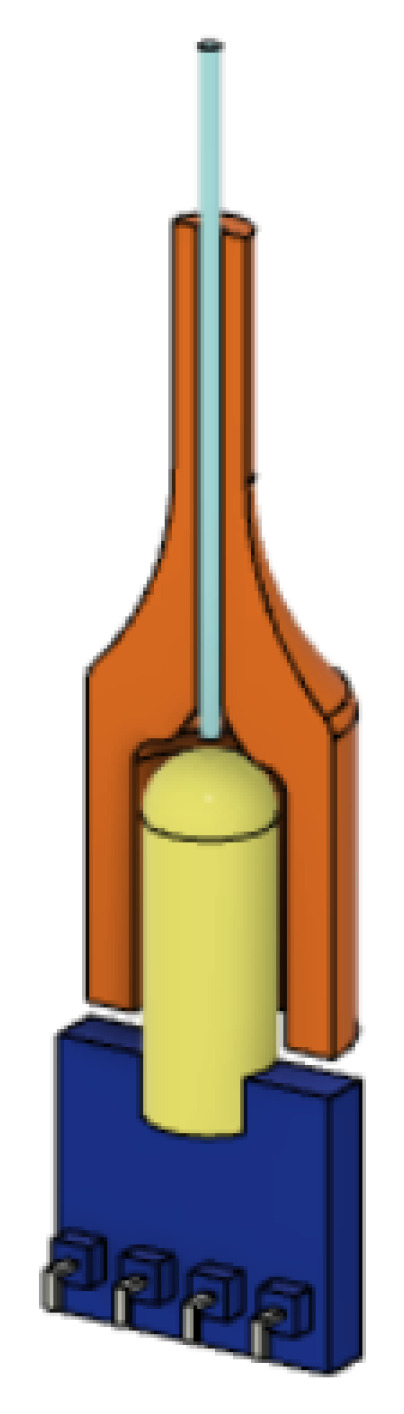
Designed optical connector (in orange) joining optical fiber and receiver PCB.

The optical joints at the interface between fibers and transmitters/receivers are obtained through a connector obtained from optimized design and 3D-printing, in order to convey the light from the source into the fiber more efficiently, and from the latter towards the receiver (see Figure 4).

Since both the actuation and sensing of the continuum manipulator are based on a pair of optical fibers of commercial PMMA—despite PMMA being rigid and its mechanical performance being suitable for the application—unexpected radial deformation and tensile response under applied actuation and external forces need to be carefully evaluated in terms of load effects on the bending measurement.

Therefore, the overall structural design of the robot has considered these issues by optimizing the geometry (i.e., layout and dimensions of the sections of the robot) to limit local strains on the optical fibers undergoing looping and pulling actions. Therefore, the design and optimization of the 3D model of the kinematic chain of the continuum manipulator are discussed in further detail below.

### 2.3. Hyper-Redundant Continuum-Robot Kinematics with Rolling Joints and Cable-Driven Actuation

**Figure 5:**
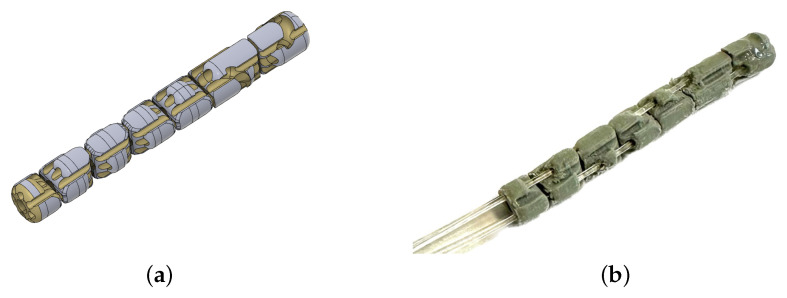
CAD-3D model of the continuum robot (**a**) compared with the real 3D-printed prototype (**b**).

**Figure 6:**
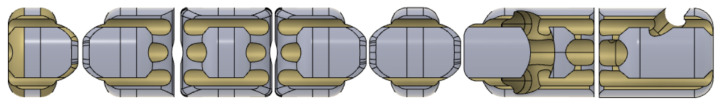
Lateral view of the continuum manipulator.

**Figure 7:**
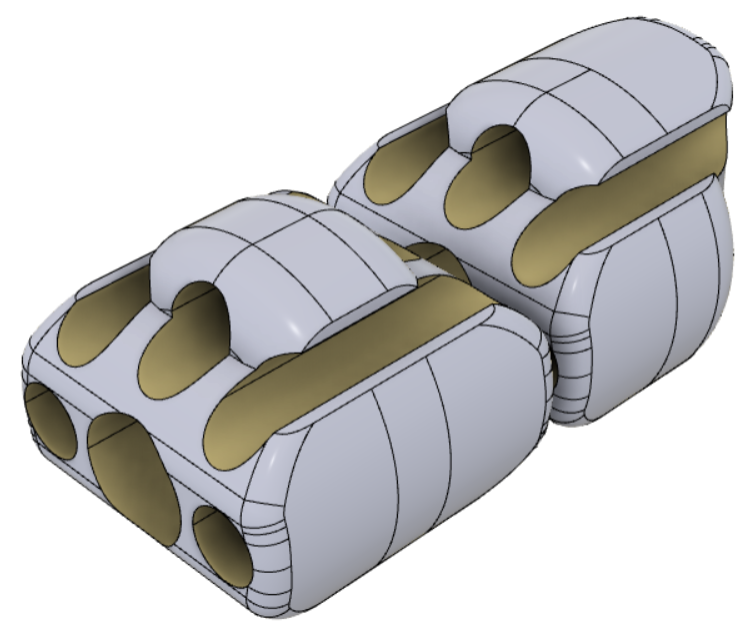
Detailed view of two consecutive sections in rolling contact within the continuum kinematics.

The manipulator is characterized by a continuum kinematics obtained through the serial connection of seven structural modules through PMMA tendons and rolling joints. The resulting structure (see Figure 5 and Figure 6) is characterized by the typical controllable compliance provided by the rolling joints (see Figure 7).

The conjoint adoption of rolling joints and tendon-driven actuation guarantees the improved motion accuracy and enhanced payload capability required by the applications of neurosurgical robotics. Moreover, the rolling-joints kinematics can overcome the limitations of standard micromanipulators—e.g., weak payload and constructive complexity—due to the fabrication of miniaturized rotational joints.

**Figure 8:**
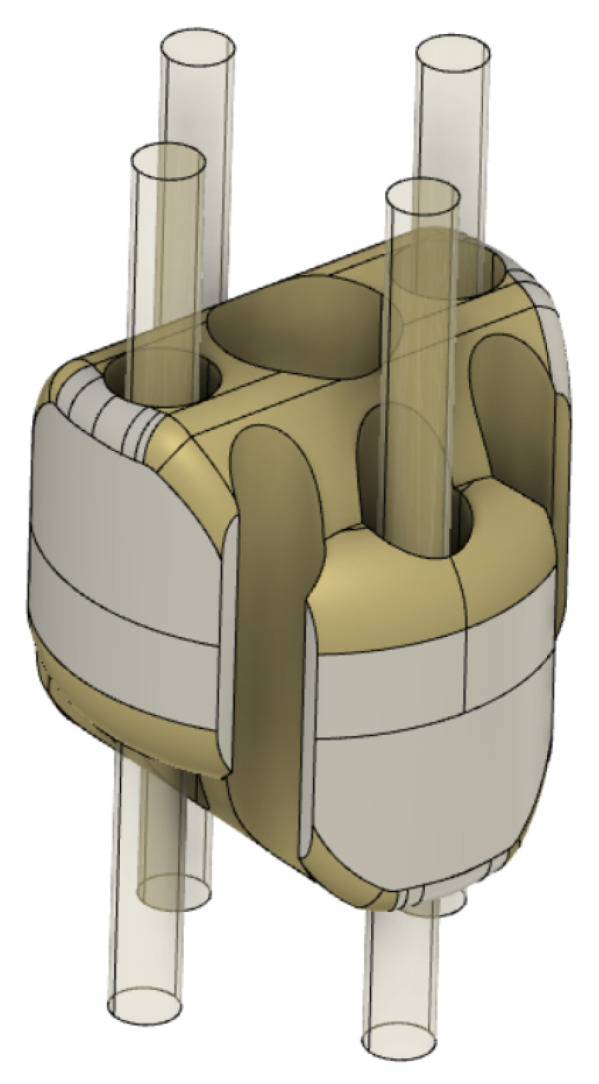
Three-dimensional (3D) view of a single section of the continuum robot. The high-transparency objects represent the PMMA fibers.

Two DoFs (distal angles of tilt and pan) can be independently controlled since the bending of the rolling-joints kinematic can be actuated through two U-shaped PMMA fibers arranged on orthogonal planes (see Figure 8). Each fiber is antagonistically actuated at the proximal position of the kinematic chain. The bending of the continuum kinematics of the manipulator is obtained—independently on each fiber plane—by alternatively pulling one of the proximal extremities of the fiber and releasing the other one. Since, in correspondence with its U-shaped extremity, the fiber is constrained to the robot body, the pulling force is transduced to the bending of the continuum kinematics, which results in the distal rotations. The main parameters of the manipulator are reported in Table 1. The main dimensions of the manipulator are compliant with the miniaturization constraints required by minimally-invasive neurosurgery. The outer diameter and length of the robot body are comparable with those of the conventional instrumentation like trocars and catheters, which are used to gain minimally-invasive access to the brain for inserting an endoscope and surgical tools.

**Figure 9:**
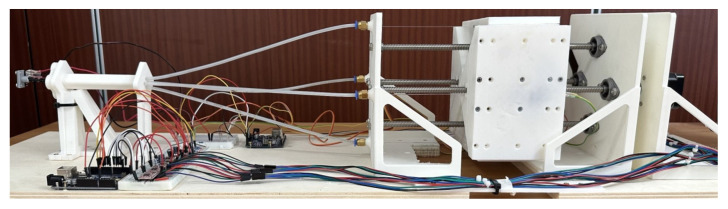
Overview of the neurosurgical robot including the continuum manipulator, actuation, and sensing modules.

**Figure 10:**
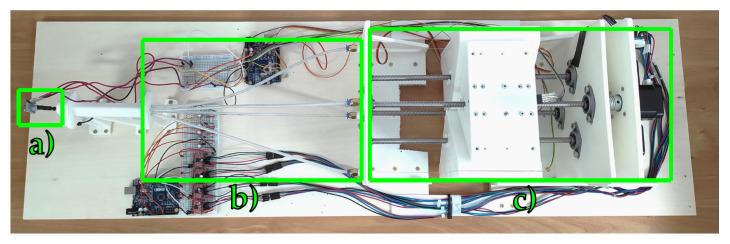
Main modules of the robotic system. (**a**) Snake-like probe. (**b**) Optical cabling and Bowden-cable transmission. (**c**) Lead-screw actuation.

The complete prototype of the robot and its main modules are displayed in Figure 9 and Figure 10. The sensing and control of the bending of the continuum manipulator (labelled by “a” in Figure 10) are achieved through the interconnection with the module “b”, where the optical channels can provide the feedback signal of the tip bending and the linear actuation forces can be transmitted through a Bowden-cable principle from the actuation module “c” to the distal extremity of the continuum manipulator. The detailed description of the function of the components of the robotic system shown in Figure 10 is associated with the discussion of the setup and experiments in Section 3.

The realization of an efficient solution for optical embedded-sensing and tendon-driven actuation has required the optimization of the 3D design and extensive testing of alternative configurations of cabling and channels within the robot body.

**Figure 11:**
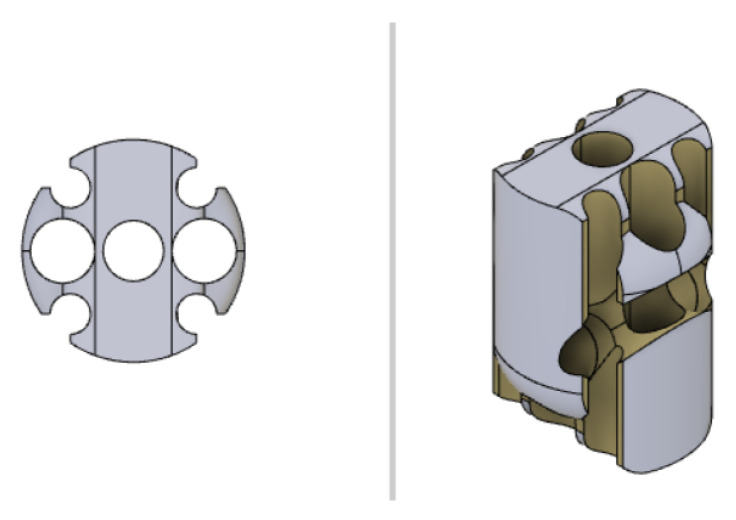
Top and 3D view of the second-last section with the U-shaped channel designed for constraining the pan-actuating fiber.

**Figure 12:**
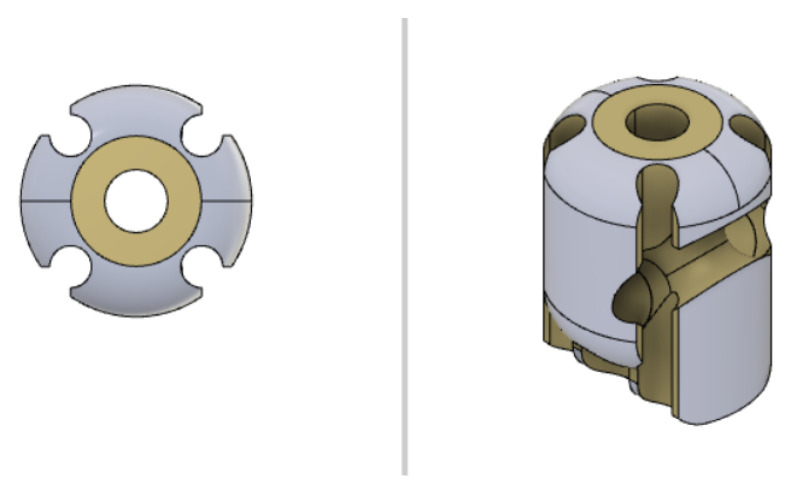
Top and 3D view of the last section with the U-shaped channel designed for constraining the tilt-actuating fiber.

Some U-shaped channels, which are fabricated within the solid geometry of the distal sections of the manipulator, are designed to obtain the closed-path of the optical fibers needed to obtain the feedback of the bending on the receiver side (see Figure 11 and Figure 12). The fibers are fixed to the robot body in correspondence with the U-shaped channels through the infusion of a layer of epoxy resin (Power Epoxy by Pattex^®^).

**Figure 13:**
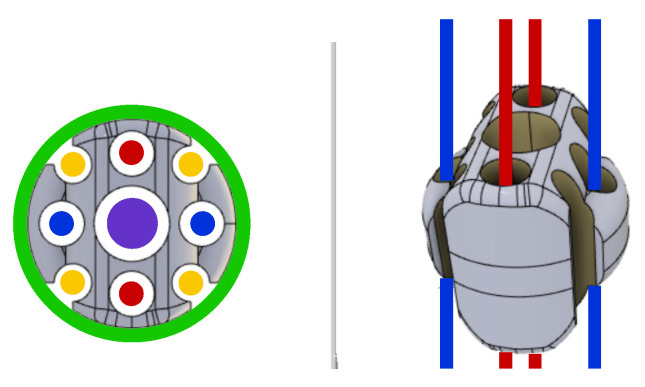
Top and 3D view of an intermediate element of the continuum kinematics.

The additional hollow space within the manipulator sections is designed to accommodate optical fibers and to host surgical tools, potentially including aspiration functions (see Figure 13). Indeed, each manipulator section has a central hole with a diameter of 1.5 mm through which to insert microsurgical instruments (highlighted in dark blue). Aspiration conduits can be obtained by exploiting both the central hole and four grooves on the lateral surface of the sections (in yellow). The realization of aspiration functions requires, for sealing purposes, an external sheath of hyper-elastic material (in green). The PMMA fibers are identified, respectively, by blue and red colors.

The curvature of the channels of the optical fibers is designed to keep the tensile stress on the fibers due to actuation tension within an acceptable range in order to avoid breaking while limiting the slackness of the fibers. Moreover, the convex protrusions, which provide the contact profiles rolling joints, are slightly flattened at a depth of 0.2 mm to improve the static bending stability of the manipulator. As a byproduct of the optimized integration of a tendon-based actuation within the robot body, both bending and torsional stiffness can be regulated by adding a pretension on the actuation tendons. Some results on optimizing the torsional stiffness of 3D-printed continuum robots are available in the literature [29,30].

The actuation module, which is in a proximal position, consists of four linear actuation units, each of which is obtained through a bipolar stepper motor NEMA17 (1.8° of step angle, holding torque 0.45 [Nm]) and a slider-screw assembly. The lead screw has a pitch of 2 mm and an external diameter of 8 mm. The adopted actuation can satisfy the payload constraint specified by the range [0.5–3] N. The microcontroller-based control board of the actuation module is implemented on Arduino UNO R3, which is interfaced to the drivers A4988 as power converters.

### 2.4. CAD-3D Modelling and 3D-Printing

The CAD-3D model of the continuum robot has been designed using dedicated software, i.e., Autodesk Fusion 360 2.0.1, and it has been 3D-printed using FDM (Fused Deposition Modelling) bottom-up technology. Each section of the continuum robot has been designed in order to accommodate the couple of fibers (one for tilt angle and one for pan angle) and to create the rolling joints at the ends of the sections themselves. Particular attention has been paid to the design of the last two sections, in which a U-shaped housing for the fibers guarantees the closing of the optical loop without compromising the rolling joints (see Figure 11 and Figure 12). A combination of both software and hardware configuration, setting up a nozzle with a diameter of 0.2 mm and a layer height of 0.05 mm, respectively, allowed an optimal resolution along 3D-printing axes. The chosen setup represents the best option, which is guaranteed by the available 3D-printer and printing materials, for detailed reproduction of the designed CAD-3D model in the fabrication phase. Every section has been 3D-printed in order to exploit the anisotropy implied in this technology, by guaranteeing that the revolution axes of the holes of each section match with the 3D-printer z-axis. The overall 3D-printing parameters for the continuum robot sections are listed in Table 2. These have been applied to 3D models using the slicer software IdeaMaker by RAISE 3D.

Although the robot prototype has been 3D-printed using commercial PLA, both the flexibility guaranteed by the additive manufacturing technologies and the availability of 3D-printable and bio-compatible polymeric material allow us to satisfy, on a new version, any constraint on bio-compatibility.

## 3. Experiments and Results Analysis

The optical sensing principle, upon which the estimation of the distal angles of tilt and pan is based, has been implemented into the real prototype obtained at the end of the phases of innovative design and the realization of the opto-mechatronic measurement system.

The robot prototype and its sensing module have been tested with a set of experiments to assess the robust estimation of the distal angles of the flexible structure of the continuum manipulator. The validation has involved the fitting of a linear regression model correlating the output voltage signals of the photodiode with the experimentally measured angles. All these experimental steps are presented and discussed further below.

### 3.1. Experimental Setup for the Experimental Characterization of Bending Sensing Module

The experimental data needed for identifying the multiple regression model are acquired from the setup depicted in Figure 9 and Figure 10. An inertial measurement unit (IMU) MPU6050 provides the ground-truth data that are used for the fitting. To this end, the IMU is fixed to the distal extremity of the continuum manipulator through an adapter that can be fitted through calibrated mechanical interference on the tip of the continuum kinematics of the manipulator. Through the initial setup of the adapter, the IMU is mechanically fixed to the robot tip with its measurement axes (pitch and yaw) aligned to the axes of distal rotations of the robot (tilt and pan). This setup assures that the IMU can provide reference data of the distal angles to be correlated, during the fitting of the regression model, to the voltage output signals of the photo-receivers.

**Figure 14:**
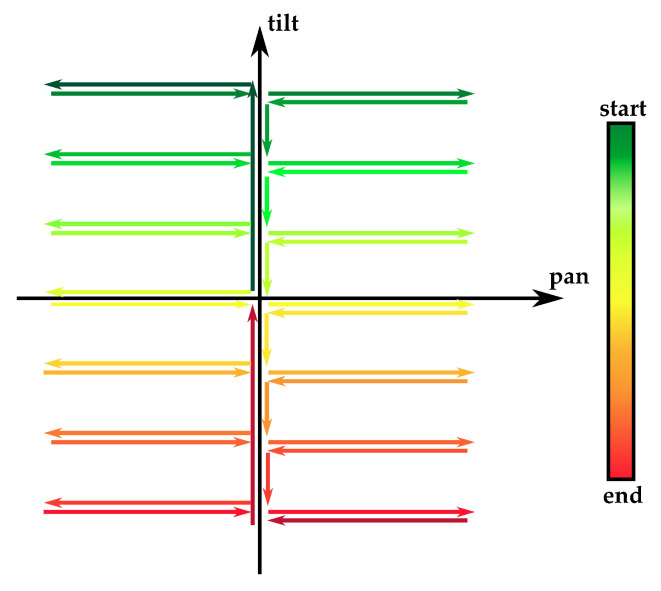
Reference trajectory used for model identification in 2-DoF space. Starting from the no-bending configuration (0° on both tilt and pan axes), the tip reaches a rotation range of about ±33° on both tilt and pan axes.

Moreover, the experimental prototype shown in Figure 9 and Figure 10 includes some force-resistive sensors which are installed, through a suitable support structure provided by a mounting plate, to estimate the tensioning of the PMMA cables during antagonistic actuation. The adoption of such force sensors is motivated by both the need for miniaturization and the accurate characterization of the measurement output and/or interaction forces. Moreover, the output signals from force sensors can be used for complementing the output signals of the photodiode in order to discriminate the rotation direction of the distal angles. A C-like firmware has been developed and loaded on the microcontroller-based control board to obtain periodic profiles of bending (see Figure 14) by driving the linear actuators, which pull the manipulator tendons while the output voltage values have been acquired.

The same firmware governs both the acquisition of force and photodiode voltage output and the low-level control law used to follow the trajectory depicted in Figure 14.

A linear least-square regression model (for each DoF) has been estimated on the basis of the experimental data sampled along the reference trajectory in Figure 14, i.e., a couple of voltage output signals from the photodiodes (associated to the two orthogonal planes in Figure 2) vs. the IMU readings of pan and tilt angles, respectively.

**Figure 15:**
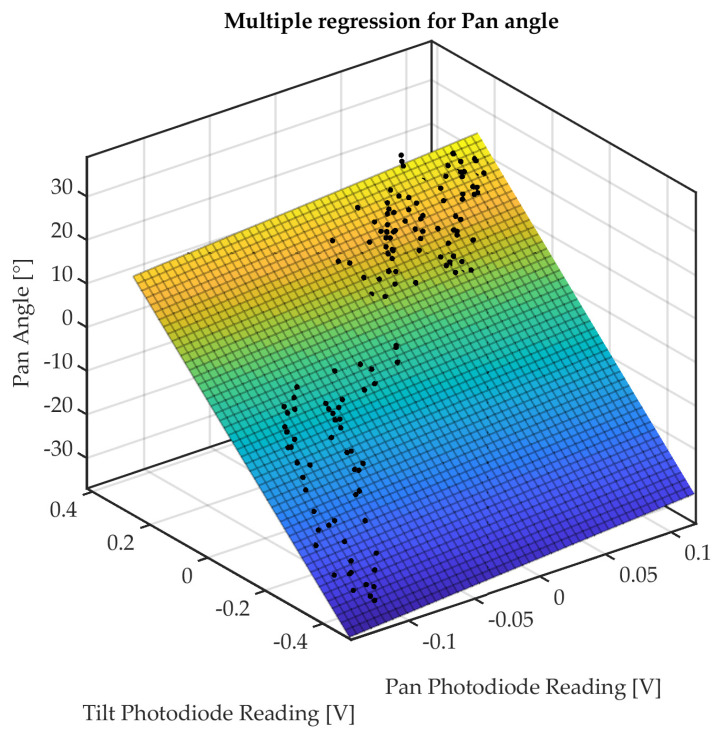
Multiple regression of pan angle.

**Figure 16:**
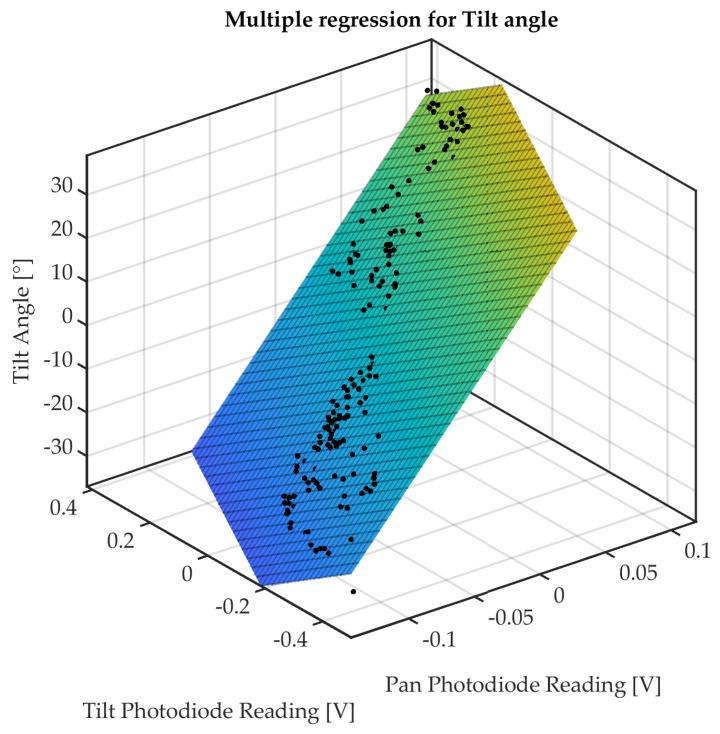
Multiple regression of tilt angle.

A multiple regression has been carried out using the Curve Fitting Toolbox of Matlab 2022b^®^, by identifying the fitting planes for both tilt and pan angles (see Figure 15 and Figure 16).

Since the attenuation of the voltage readings of the photodiodes cannot discriminate between the rotation direction for each axis, the firmware has been deployed on the embedded control board in order to change the sign of the photodiodes readings accordingly with the direction of movement of the tip, which can be derived from FSRs.

The linear fitting function is

(1) $$f\left(x,y\right)=p_{00}+p_{10}x+p_{01}y\;,$$

whose parameters, which are estimated for tilt and pan angles, are listed in Table 3. The output voltages acquired from tilt and pan photodiodes are denoted by the regression variables *x* and *y*, respectively.

The RMSE obtained for tilt and pan angles (values of 6.946° and 6.284°, respectively) can provide a performance index of the estimation from the linear fitting model. These values are within the range of the RMSE obtained in state-of-the-art optical shape sensing for tendon-driven flexible robotic manipulators (see [31]). A linear fitting model can be considered the best option whenever the digital acquisition and elaboration for bending estimation are implemented on a microcontroller-based embedded control board.

### 3.2. Analysis of the Model Accuracy over Experimental Patterns of Motion

The experimental tests have been carried out on the optical measurement system to obtain a global evaluation of the estimation performance whenever the manipulator is bent over a 2-DoF plane spanning the operating ranges of tilt and pan angles. Moreover, within this plane, a specific pattern of bending, which is associated with a worsening of the bending estimation, has been identified.

**Figure 17:**
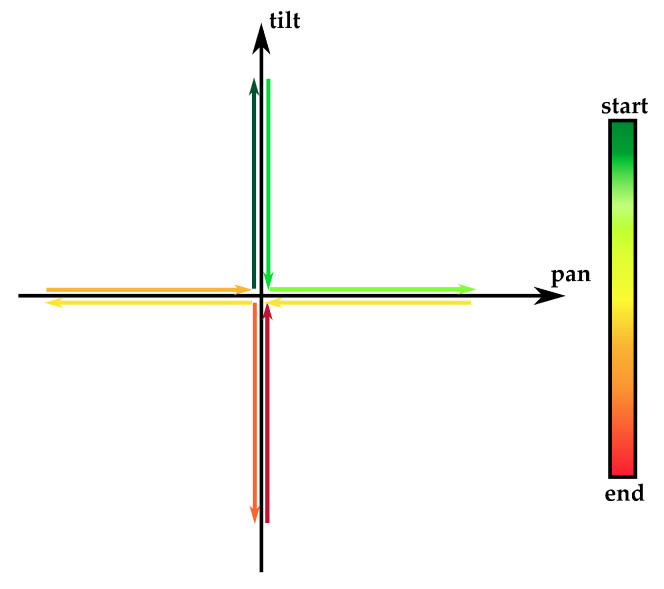
Experimental motion pattern. Starting from the no-bending position (0° on both tilt and pan axes), the sequence of the arrows goes from dark green to dark red.

Indeed, in further experiments, the manipulator body is bent just along the coordinated axes (see Figure 17), representing the worst case for the measurement performance, in which, for every sample, at least one fiber is not fully stretched. To evaluate the performance of the prediction in this worst-case, the multiple linear regression model (1) has been tested on this new dataset, in which the pre-processing was the same as that used in the phase of fitting. As shown in Table 4, the RMSE has remained almost the same in the case of the pan angle, while it doubled in the tilt angle.

**Figure 18:**
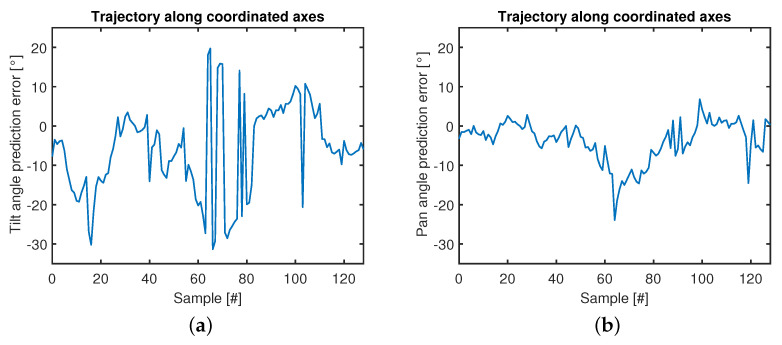
Tilt (**a**) and pan (**b**) angles prediction errors. Trajectory along coordinated axes.

The ground-truth data collected by the IMU can be used as reference values to calculate the measurement errors of the optical module (see Figure 18). The calculated values of RMSE and mean error, which are reported in Table 4, can be used to estimate the accuracy of the embedded sensing module, through some robust indices of the measurement performance.

The results show that the bending measurement performance, which is achieved through embedded fibers and standalone opto-electronic modules in nominal conditions within the region in Figure 14, is acceptable since this result is obtained in a miniaturized and flexible manipulator complying with MRI-compatibility requirements.

The applicability and performance of the sensing principle based on embedded optical fibers, which has been successfully implemented in a flexible and hyper-redundant kinematic chain of a continuum manipulator, can be further enhanced and extended to a general class of miniaturized shape-sensing systems.

Further analysis is needed to identify suitable strategies to improve the bending measurement performance, e.g., through the optimization of the structural design of the continuum manipulator by combining the number of elements and directions of the rotation axes of consecutive sections within the rolling-joints kinematics to reduce both the mechanical compliance and tendon slackness. Another important aspect to be investigated, through a specific set of experimental tests, is the potential behavior of the optical fiber-receiver assembly characterized by non-negligible sensitivity thresholds, especially in the measurement of the tilt angle.

Both the tendon slackness and the intrinsic compliance of the kinematic chain, which limit the measurement performance along specific bending motion, can be potentially compensated through alternative control strategies, i.e., for fine regulation of the tension of the tendons actuating the bending DoF of the robot, as well as pre-programmed motion avoiding the worst-case trajectory shown in Figure 17.

### 3.3. Derivation of a Kinematic Model of the Continuum Robot

The test-rig of the continuum manipulator has been used for the experimental identification of the direct kinematic functions correlating the linear actuation inputs on the proximal extremity of the kinematic chain with the distal DoF (tilt and pan angles) at the distal end of the robot.

The two angles are actuated at increments of one motor step in order to enclose into the set of experimental points a range of [−30°, 30°] for tilt and pan angles. The bending angles in response to the actuation inputs are measured by the IMU and acquired on the microcontroller board. Therefore, the measured responses angle vs. linear displacement have been fitted through the linear function,

(2) $$\begin{array}{c} f\left(x\right)=p_{1}x+p_{2}\;, \end{array}$$

which gives the expression of the angular output with regard to the input *x*, i.e., the linear displacement actuated for tilt and pan, respectively. The functions parameters are reported in Table 5.

Each bending motion has been repeated five times for the two DoFs. Through a statistical analysis of the experimentally-identified kinematic functions, it is possible to quantify the controllability of the tip bending.

**Figure 19:**
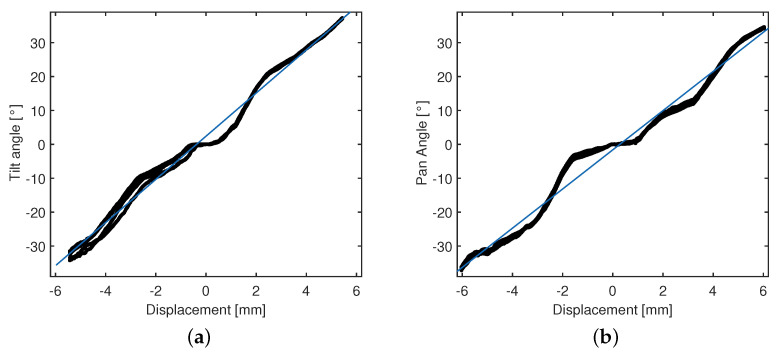
Experimental identification of the kinematic responses under tendon-driven actuation. Tilt (**a**) and pan (**b**) angles. Experimental data (in black) and linear fitting curves (in blue).

The goodness-of-fit of the linear functions in Figure 19 has been estimated through the parameters reported in Table 6.

The achieved values of $R^{2}$ show that some linear functions can be adopted for identifying a kinematic model of the continuum robot in the range [−30°, 30]. Moreover, the linearity of the kinematic response of the robot is appealing for the controllability of the tip bending by adopting a tendon-driven kinematics actuated by two couples of actuators in antagonistic configuration.

However, it is noteworthy that, for small linear displacements around the initial position, the kinematic responses are characterized by a reduced sensitivity, which may arise from both the tendon slackness and the fabrication tolerances of the kinematic chain (see Figure 19).

### 3.4. Experimental Evaluation of the Closed-Control Performance in Coordinated Tilt-Pan Rotations

The measurement performance, which is achieved through the optical sensing module of the continuum robot, has been evaluated on the basis of the tests and performance indices reported in Section 3.2.

For a complete assessment of the closed-loop control performance resulting from the features of the sensing module and actuated kinematics of the continuum manipulator, the accuracy and repeatability of the controlled bending of the robot tip have been estimated through an additional set of repeated tests of the regulation of tilt and pan angles to the set-point values of 30°.

The regulation is implemented within the closed-loop control system by providing the controller with the feedback signals of the bending angles measured through the optical sensing module. The angular velocities of the stepper motors are controlled through a switching law; the switching condition is based on the measured values corresponding to the desired set-points of the bending angles.

During the rising phase of bending, the two pairs of motors are antagonistically commanded at a velocity (in absolute value) of 200 °/s; in correspondence to the measured values of 30° for each bending angle, the rotation velocities are switched to null values.

The continuum kinematics, under tendon-driven linear actuation and a closed-loop controller, can achieve both reduced steady-state errors and good repeatability of the bending trajectory.

Table 7 reports the mean and standard deviation of the steady-state errors calculated on the motion profiles. Both the estimation of the control performance indices and the plot of the experimental profiles are obtained on the basis of the real measurements provided by the IMU. 

**Figure 20:**
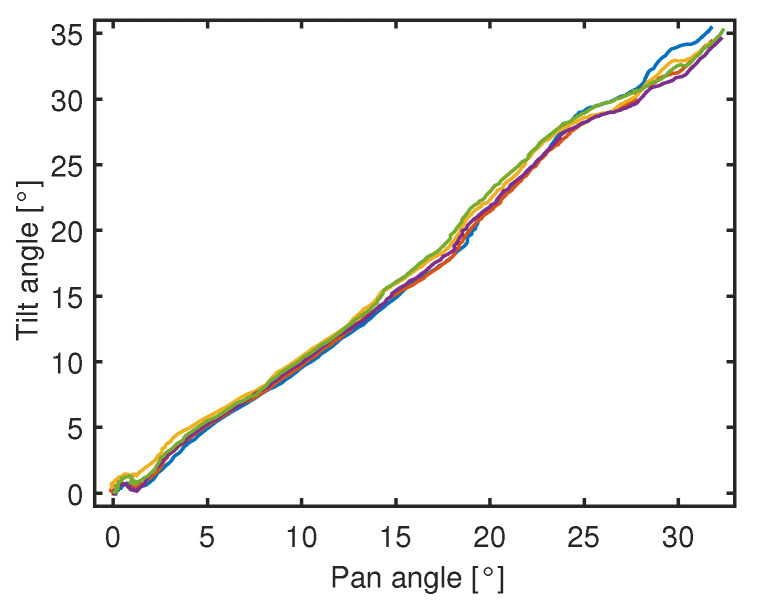
Coordinated bending of the robot tip: repeated tests of regulation of tilt and pan angles (denoted by different colors).

**Figure 21:**
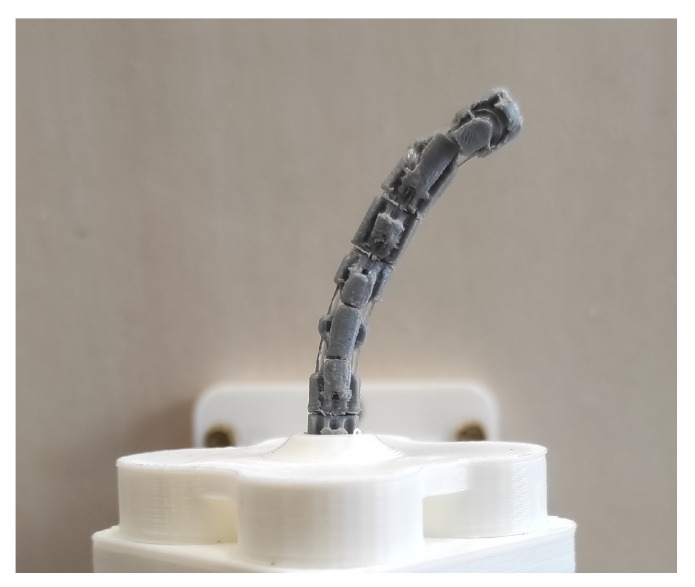
Continuum robot in bending configuration.

The accurate positioning around the desired final point (30°, 30°) and the good repeatability of the bending profiles can be confirmed through their representation on the DoF plane in Figure 20. A photo of the robot prototype in bending configuration is provided in Figure 21.

## 4. Conclusions and Future Work

This paper has presented a novel prototype of a continuum manipulator, which can be integrated with active bending catheters for supporting the automation of neurosurgical tasks. The overall specifications of the mechatronic design developed are well-suited for the improvement of the dexterity and positioning of the distal extremity of the robot, e.g., during the insertion and manipulation of microsurgical instruments through the tubular body of the manipulator.

Moreover, a distinctive feature of such a continuum robot is the optical sensing components, which are embedded into the robot’s structure to support the implementation of the closed-loop control laws of the distal bending and body curvature of the robot. The measurement performance of the embedded sensing solution has been verified by experimental tests and, after accurate calibration through a linear regression model, the results have been compared with ground truth data provided by an IMU sensor.

The experimental tests have been directed towards the validation of the robustness of the measurement of the distal angles (of tilt and pan at the tip of the continuum manipulator) via embedded optical-fiber sensors in continuum manipulators exploiting cable-driven actuation. The experimental validation has involved the evaluation of the sensing and control performance of the robot during tip bending tasks.

In conclusion, the actual prototype of a hyper-redundant and flexible manipulator can be further developed and implemented in a control system targeted at the neurosurgical applications of biomedical robotics with increased dexterity and accurate positioning, in fulfillment of some constraints on the safe interaction and miniaturization of the robot’s structure.

To increase the rigidity of the kinematic chain in order to reduce the measurement errors, especially for the tilt angle and in the presence of environmental interaction, future prototypes will be potentially controlled in terms of mixed bending/force closed-loop by exploiting the feedback of the pulling forces on the robot tendons, which is made available by the force sensor installed on the robotic system. Moreover, more structured models, which can describe nonlinearity and sensitivity thresholds, will be considered to improve both the bending estimation and the resulting control performance.

## Figures and Tables

**Table 1:** Main geometrical and physical parameters of the continuum manipulator.

Main Geometrical and Physical Parameters of the Continuum Manipulator	
DoF	2
Range of distal bending angles	±45°
Weight	1.0 g
Shaft diameter	5.5 mm
Length	43 mm

**Table 2:** Endoscope 3D printing parameters.

Main 3D Printing Parameters for Continuum Manipulator	
Material	PLA (Raise 3D Premium)
3D Printer	Raise 3D Pro 2
Layer height	0.05 mm
Nozzle diameter	0.2 mm
Wall line count	2
Infill density	15%—Grid type
Support	None
Build plate adhesion	Raft
Extrusion temperature	205 °C
Print speed	50 mm/s

**Table 3:** Output of the multiple linear regression for tilt and pan angles: surface fitting data.

Parameter	Tilt Angle	Pan Angle
*p*_00_	5.504	2.442
*p*_10_	355.8	23.52
*p*_01_	−46.85	72.13
*R*-squared	0.8538	0.823
RMSE	6.946	6.284

**Table 4:** Measurement performance indices for the trajectories along coordinated axes.

Index	Tilt Angle	Pan Angle
RMSE	12.75	6.44
Mean error	−5.59	−3.76

**Table 5:** Parameters of the identified kinematic functions.

Index	Tilt Angle	Pan Angle
$p_{1}$	6.366	5.78
$p_{2}$	2.407	−1.613

**Table 6:** Evaluation of the goodness-of-fit of the linear kinematic functions.

Index	Tilt Angle	Pan Angle
RMSE	2.26	2.84
$R^{2}$	0.987	0.981

**Table 7:** Steady-state errors over repeated tests of tip bending regulation.

Index	Tilt Angle	Pan Angle
Mean	−3.02	−1.51
Standard deviation	0.48	0.19
